# 1759. Risk Factors for OPAT-Related Adverse Drug Events: A Case-Control Study

**DOI:** 10.1093/ofid/ofac492.1389

**Published:** 2022-12-15

**Authors:** Elysia Burke, Desiree Croteau, Lauren Dutcher, Keith W Hamilton, Kathleen Degnan, Tiffany Lee, Steve Saw, Sonal Patel, Shawn Binkley, Vasilios Athans

**Affiliations:** Hospital of the University of Pennsylvania, Philadelphia, Pennsylvania; Hospital of the University of Pennsylvania, Philadelphia, Pennsylvania; University of Pennsylvania Perelman School of Medicine, Philadelphia, Pennsylvania; University of Pennsylvania Perelman School of Medicine, Philadelphia, Pennsylvania; University of Pennsylvania Perelman School of Medicine, Philadelphia, Pennsylvania; Hospital of the University of Pennsylvania, Philadelphia, Pennsylvania; Hospital of the University of Pennsylvania, Philadelphia, Pennsylvania; Hospital of the University of Pennsylvania, Philadelphia, Pennsylvania; Hospital of the University of Pennsylvania, Philadelphia, Pennsylvania; Hospital of the University of Pennsylvania, Philadelphia, Pennsylvania

## Abstract

**Background:**

Outpatient parenteral antimicrobial therapy (OPAT) offers numerous clinical advantages, though adverse drug events (ADEs) are a common and potentially preventable challenge that may contribute to 30-day readmissions and other negative outcomes. In January 2021, our OPAT program began documenting all significant ADEs using an electronic template. The purpose of this study was to characterize significant OPAT ADEs and to identify potential risk factors for their development.

**Methods:**

Outpatient parenteral antimicrobial therapy (OPAT) offers numerous clinical advantages, though adverse drug events (ADEs) are a common and potentially preventable challenge that may contribute to 30-day readmissions and other negative outcomes. In January 2021, our OPAT program began documenting all significant ADEs using an electronic template. The purpose of this study was to characterize significant OPAT ADEs and to identify potential risk factors for their development.

**Results:**

Cumulative ADE incidence was 11%, and median time-to-ADE was 13 days after discharge. During the study period, 54 ADE patients vs. 100 control patients were identified. The most common ADEs attributed to OPAT were kidney injury (50%), rash (10%), and leukopenia (9%) (Table 1). Most ADEs resulted in an OPAT regimen change (33%), dosage adjustment (29%), or early cessation of OPAT (21%) (Table 2). In the final logistic regression model, receipt of vancomycin, use of empiric therapy for culture-negative infection, and OPAT duration ≥ 28 days were associated with increased ADE risk, whereas African American race and receipt of ceftriaxone were protective (Table 3).

**Figure:**
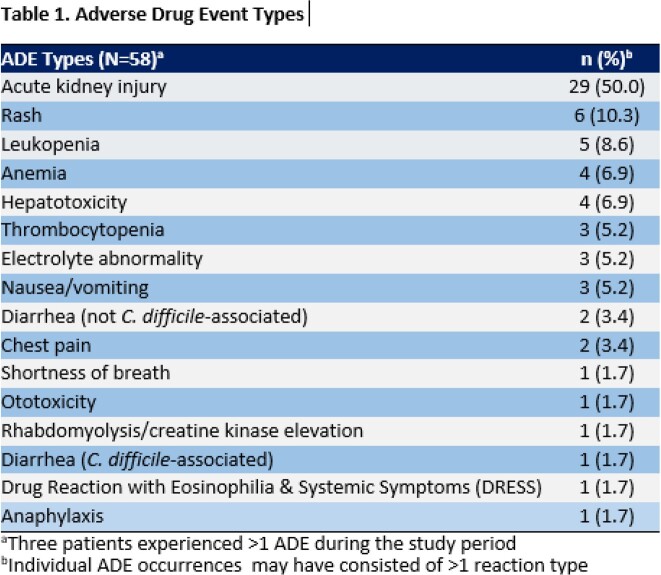

**Figure:**
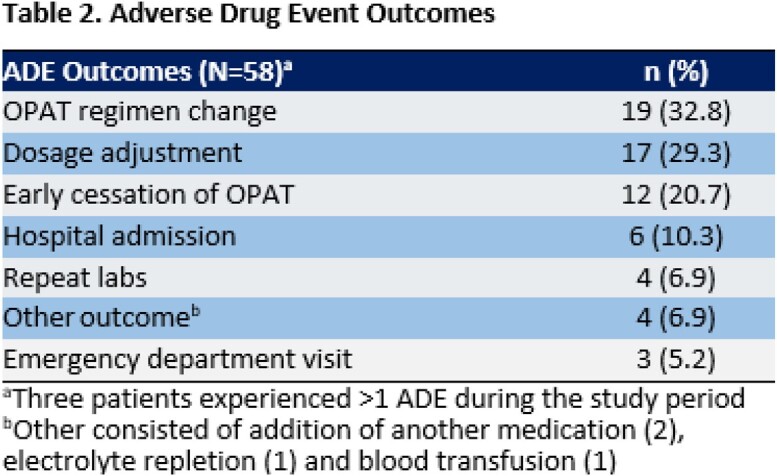

**Figure:**
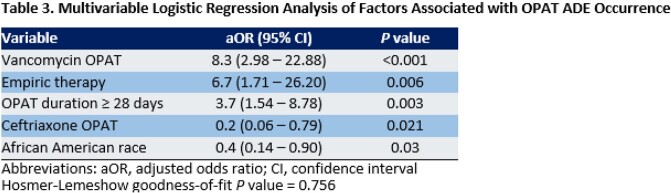

**Conclusion:**

Several modifiable risk factors may increase the likelihood of an ADE during OPAT and should be carefully considered prior to hospital discharge. Based on these data, OPAT programs should consider employing vancomycin alternatives, diagnostic stewardship, and strategies to minimize duration of therapy.

**Disclosures:**

**Kathleen Degnan, MD**, Gilead: Grant/Research Support.

